# A novel TSC2 variant cosegregating with TSC in the family: A case report

**DOI:** 10.1097/MD.0000000000041576

**Published:** 2025-02-28

**Authors:** Jianwei Cao, Chuwen Zeng, Longhui Shao, Meiling Liu, Ze’e Wu, Xiaowen Zhang, Mingxing Liu, Runyu Zhong, Kaijun Zheng, Jialong Chen

**Affiliations:** a Kangyi VIP Outpatient Clinic, Zhongshan People’s Hospital, Zhongshan, Guangdong, China; b Department of Environmental and Occupational Health, School of Public Health, Guangdong Medical University, Dongguan, China; c Dongguan Key Laboratory of Environmental Medicine, School of Public Health, Guangdong Medical University, Dongguan, China; d Department of Pediatrics, Zhongshan People’s Hospital, Guangdong, China; e Department of Rehabilitation, Zhongshan People’s Hospital, Guangdong, China.

**Keywords:** familial aggregation, heredity, novel mutation site, TSC2 gene, tuberous sclerosis complex

## Abstract

**Rationale:**

Tuberous sclerosis complex is a multisystem genetic disorder caused by variant of *TSC1* or *TSC2*, which were defined as an independent diagnostic criterion for TSC.

**Patient concerns:**

We present a novel hereditary variant in a family. The family showed a phenomenon of familial aggregation in the Tuberous sclerosis complex.

**Diagnoses:**

The proband had the c.3974del (exon 33) (p.Gly1325Alafs*58) loss of heterozygosity frameshift in the TSC2 gene (chr16), which was 1 base deletion on the coding sequence of TSC2, leading to a frameshift mutation. Moreover, the novel variant occurred in the grandchildren (generation 3) also can be detected in the grandparental (generation 1) and parental (generation 2).

**Interventions:**

The proband had taken antiepileptic drugs (oxcarbazepine [30 mg/(kg·day)], depakine [28 mg/(kg·day)], levetiracetam [38 mg/(kg·day)], and lamotrigine [2 mg/(kg·day)]) and performed a right parietal resection of the epileptic lesion.

**Outcomes:**

The treatment received by the proband was ineffective.

**Lessons:**

The novel gene mutation sites to be found provide more research entry points for genetic diagnosis, providing new clinical data for tuberous sclerosis complex research.

## 1. Introduction

Tuberous sclerosis complex (TSC) is a rare autosomal dominant genetic disease characterized by multiorgan hamartomas.^[1,2]^ Cerebral cognitive abnormality is the most common manifestations of the disease, as well as neurological features include seizure, autism, and intellectual disability.^[3]^ The earliest manifestations of TSC were skin lesions (80% of patients) and seizure (75% of patients). The hallmark skin lesions of TSC include angiofibroma, shagreen patch, fibrous cephalic plaque, hypomelanotic macules, and ungual fibromas.^[4,5]^ TSC is caused by mutations of either of 2 genes, *TSC1* (encoding hamartin) and *TSC2* (encoding tuberin), which were defined as an independent diagnostic criterion for TSC in the latest TSC diagnostic consensus.^[6]^ *TSC1* and *TSC2* are responsible for regulating the normal expression of the mechanistic target of rapamycin complex 1 (mTORC1).^[7]^ However, up to 10% to 15% of patients with clinically definite diagnosed TSC were negative for gene test and found no mutations, indicating that gene test may not completely rule out the diagnosis for TSC.^[8,9]^ Finding more undiscovered gene mutation locations expands the research opportunities for genetic diagnostics.

Here, we report a family with TSC. Whole exome sequencing and Sanger sequencing were used and genetically analyzed the family. Proband had the c.3974del (exon 33) (p.Gly1325Alafs*58) loss of heterozygosity frameshift in the TSC2 gene (chr16), which was a novel variant and also can be found in the female members of her family. Finding out the kind and heritability of the new variant in the proband and her family is helpful for prenatal diagnosis.

## 2. Materials and methods

### 2.1. Subjects

**Figure 1.**
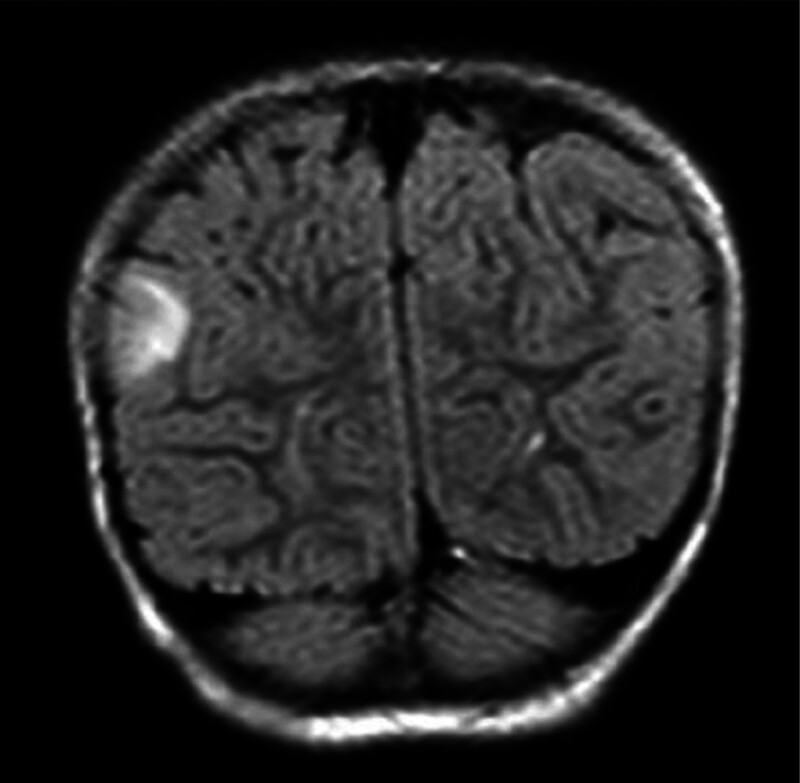
Brain MR features of the proband. MR = Mendelian randomization.

**Figure 2.**
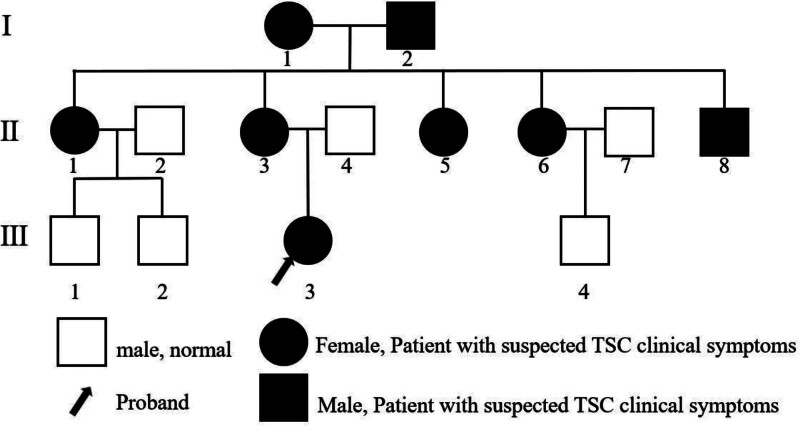
Immediate relatives of proband. TSC = tuberous sclerosis complex.

The studies involving human participants were reviewed and approved by the Zhongshan People’s Hospital, Zhongshan, Guangdong. The participants provided their written informed consent to participate in this study. The proband was female, 6 years old. No abnormalities were reported in her birth history and growth history. She had seizures twice at 19 months after birth and preformed an involuntary tremor of the jaw with drooling after 3 months. A video electroencephalogram showed diffuse and non-lateral epileptiform discharge. Brain MR showed multiple abnormal signals in the right parietal lobe, left frontal cortex and subcortical cortex, indicating the possibility of focal cortical dysplasia (FCD) and dysembryoplastic neuroepithelial tumors (Fig. 1). She had taken antiepileptic drugs (oxcarbazepine [30 mg/(kg·day)], depakine [28 mg/(kg·day)], levetiracetam [38 mg/(kg·day)], and lamotrigine [2 mg/(kg·day)]) and performed a right parietal resection of the epileptic lesion, but the treatment was ineffective. And the postoperative brain MR indicated the possibility of the right parietal lobe FCD with ganglioglioma (WHO I; FCD Ⅲb). All her immediate relatives were included in this study (Fig. 2).

### 2.2. Experimental methods

#### 2.2.1. Clinical information collection

Proband and her relatives were reviewed medical records, inquired about past medical history and family history, and underwent a standardized physical examination and accessory examinations.

#### 2.2.2. Peripheral blood genomic DNA extraction

After communicating with the guardians of the children and signing the informed consent, 2 mL of venous blood was collected from the children and their parents, and ethylenediaminetetraacetic acid added for anticoagulation treatment.

#### 2.2.3. Whole exome sequencing

Probe hybridization was used to capture the exon regions and adjacent intron regions of 20,000 genes related to genetic diseases. The capture probes were designed by IDT Corporation of the United States.

#### 2.2.4. Sanger sequencing

The Illumina Novaseq 6000 next-generation sequencer was used for sequencing detection. The average sequencing depth of the samples reached 100× to 200×, and the coverage of the 10× target region reached more than 99%. Sequencing raw data uses the BWA (v0.7.10-r789) tool to compare the effective data to the genome (hg19), uses the GATK (v4.0.6.0) tool to obtain insertion and deletion mutations, and then uses the ANNOVAR (v2018Apr16) tool for gene mutation note. Whole-exome-wide copy number variation was analyzed by self-written software.

#### 2.2.5. Diagnostic criteria of TSC

On genetic diagnostic criteria, the identification of either a TSC1 or TSC2 pathogenic mutation in DNA from normal tissue is sufficient to make a “definite diagnosis of TSC.” On clinical diagnostic criteria, patients with 2 major features or 1 major feature with ≥2 minor features or with a pathogenic variant fulfilled the “definite diagnosis of TSC; patients with either 1 major feature or ≥ two minor features met “possible diagnosis of TSC.”^[6]^

## 3. Results

Proband and her relatives were reviewed medical records and underwent physical examinations (Fig. 2). The proband and most of her relatives had the typical clinical symptoms of TSC (Table 1). Proband, I_1_, I_2_, and II_6_ have 1 major TSC clinical feature (hypomelanotic macules) and were classified as “possible diagnosis of TSC.” II_1_ has 2 major TSC clinical feature (facial angiofibromas and shagreen patch) and was classified as “definite diagnosis of TSC.” And II_3_ has 2 major TSC clinical feature (hypomelanotic macules and shagreen patch) and was classified as “definite diagnosis of TSC.”

**Table 1:** The clinical symptoms of relatives of proband.

	Proband	I_1_	I_2_	II_1_	II_3_	II_5_	II_6_	II_8_
Age	6	58	58	33	31	29	26	25
Simple partial seizure generalized	**+**	**+**	**−**	**−**	**−**	**−**	**−**	**−**
	**−**	**−**	**−**	**−**	**−**	**+**	**−**	**−**
Intellectual disability	Moderate	**−**	**−**	**−**	**−**	Moderate	**−**	**−**
Mental disorder	**−**	**−**	**−**	**−**	**−**	**+**	**−**	**−**
Facial angiofibromas	**−**	**−**	**−**	**+**	**−**	**−**	**−**	**−**
Hypomelanotic macules	**+**	**+**	**+**	**−**	**+**	**−**	**+**	**−**
Shagreen patch	**−**	**−**	**−**	**+**	**+**	**−**	**−**	**−**
Piebaldism	**−**	**−**	**−**	**−**	**+**	**−**	**−**	**+**
Structural abnormalities of the cerebral cortex	**+**	**−**	**−**	**+**	**+**	**−**	**−**	**−**

**Figure 3.**
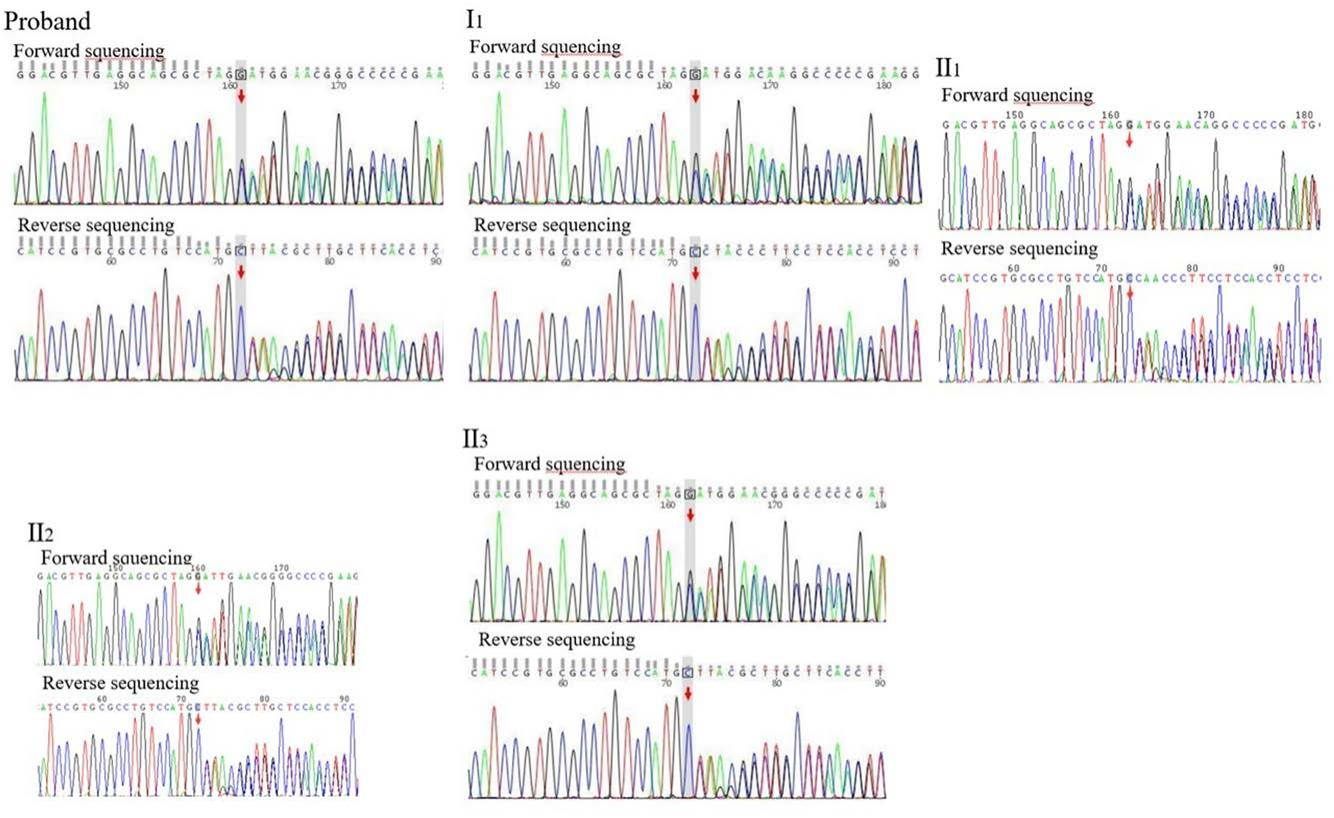
The coding sequence of TSC2 has a single base deleted (the arrow indicates the site of deleted base).

The genomic DNA of the proband was subjected to whole-exome capture and sequencing, as well as base identification of all sequencing fragments (Fig. 3). Sequencing results showed that proband had the c.3974del (exon 33) (p.Gly1325Alafs*58) loss of heterozygosity in the TSC2 gene (chr16). The mutation was 1 base deletion on coding sequence of *TSC2*, leading to a frameshift mutation. Loss of protein function could theoretically be caused by nonsense-mediated mRNA degradation or premature termination of the encoded amino acid sequence. Moreover, the clinical symptoms caused by the mutation are consistent with the clinical phenotype and genetic pattern of the subject. Collectively, these results suggested that the mutation was rated as suspected pathogenic or pathogenic.

Proband and her female family members (mother, aunts, and grandmother) showed different degrees of typical clinical manifestations of TSC, indicating that the rare phenomenon of familial aggregation. Intriguingly, the same gene fragments in the relatives were sequenced via Sanger sequencing, showing that the female members all had the c.3974del (p.Gly1325Alafs*58) loss of heterozygosity frameshift in the TSC2 gene (chr16), except for II_6_. Notably, the mutation occurred in the grandchildren (generation 3) can be detected in the grandparental (generation 1) and parental (generation 2). These data indicated that the frameshift mutation was steadily hereditary.

The mutation and its pathogenicity have not been reported in the literature. And the mutation has not been reported to be associated with TSC in the LOVD, ClinVar, OMIM, HGMD, and gnomAD, suggesting that it was a novel mutation.

## 4. Discussion

TSC is mainly caused by mutations in *TSC1* and *TSC2*, which encodes hamartin protein and tuberin protein, respectively. The formation of hamartin–tuberin is able to suppress the activity of mTORC1, regulating cell growth and proliferation under a normal state.^[10,11]^ The mutations in *TSC1* or *TSC2* induce hyperactivity of mTORC1, promoting abnormal cell growth and suppressing autophagic cell death.^[12,13]^

Both TSC1 and TSC2 mutations were nearly uniformly distributed in their protein-coding regions. TSC1 and TSC2 have more than 1000 gene mutation sites and 8 different types of mutations. Among all TSC mutations, *TSC2* accounted for about 70%, *TSC1* accounted for about 20%, and about 10% had no mutation identified (NMI) in *TSC1/TSC2*.^[14]^ I_2_ and II_6_ have not been able to detect the cause of pathogenicity, though they had 1 major TSC clinical feature and were classified as “possible diagnosis of TSC.” This may be due to the inability of current gene test technologies to detect the transcription silencing caused by epigenetic modification, the possible presence of TSC3 gene, and the mutations in chimera, introns or regulatory regions.^[15–17]^

Current studies have confirmed that the patients with mutations in *TSC2* have systemic multisystem involvement, compared with mutations in *TSC1.*^[18]^ In terms of neurological manifestations, the patients with mutations in TSC2 showed higher levels of seizure, autism and intellectual disability than did the patients with mutations in *TSC1*, and the symptoms of seizure are more severe. Moreover, the patients with mutations in *TSC2* also showed higher levels of clinical manifestations of cutaneous, oral, ocular than did the patients with mutations in *TSC1*.

TSC is a multiorgan and multisystem genetic disorder that imposes considerable chronic disease burden on patients and their families, with considerable cost and detriment to quality of life.^[19]^ Angiomyolipoma, subependymal giant cell astrocytoma, lymphangiomyomatosis and complication are the leading causes of premature death in TSC.^[20]^ However, there is still no specific treatment for TSC, and comprehensive management is mainly symptomatic treatment and the discovery of treatable symptoms or complications. Everolimus and rapamycin, as main mTOR pathway inhibitors, have greater oral bioavailability and favorable pharmacokinetics.^[21,22]^ Therapeutic effect of Everolimus and rapamycin is effective on tumors, but remains to be studied on neurological symptoms.^[23]^ The prevalence of epilepsy in patients with TSC approximates 75% to 90% across the lifetime, and it is usually medically refractory epilepsy.^[24]^ The patients with medically refractory epilepsy still have seizures after taking mTOR inhibitors treatment, such as proband and I_1_. Therefore, early identification and management is necessary, optimizing favorable outcomes in family of patients.

In the study, a steadily hereditary TSC mutation was found in a family, enriching the TSC mutation spectrum in China. Moreover, the female family members with the same gene mutation site have different clinical manifestations, providing new clinical data for TSC research.

## Author contributions

**Data curation:** Jianwei Cao, Chuwen Zeng, Longhui Shao, Meiling Liu.

**Formal analysis:** Ze’e Wu.

**Funding acquisition:** Jianwei Cao, Kaijun Zheng.

**Investigation:** Jialong Chen, Chuwen Zeng, Kaijun Zheng, Ze’e Wu.

**Methodology:** Longhui Shao, Meiling Liu.

**Project administration:** Jialong Chen, Jianwei Cao.

**Resources:** Jianwei Cao, Longhui Shao.

**Software:** Ze’e Wu, Runyu Zhong.

**Supervision:** Meiling Liu.

**Visualization:** Xiaowen Zhang, Mingxing Liu, Runyu Zhong.

**Writing – original draft:** Jialong Chen, Chuwen Zeng, Longhui Shao.

**Writing – review & editing:** Jianwei Cao, Kaijun Zheng, Xiaowen Zhang, Mingxing Liu.
